# Development of Home Mechanical Ventilation in Poland in 2009–2019 Based on the Data of the National Health Fund

**DOI:** 10.3390/jcm11082098

**Published:** 2022-04-09

**Authors:** Małgorzata Czajkowska-Malinowska, Kinga Bartolik, Jacek Nasiłowski, Aleksander Kania

**Affiliations:** 1 Department of Lung Diseases and Respiratory Failure, Centre of Sleep Medicine and Respiratory Care, Kuyavian-Pomeranian Pulmonology Centre, 85-326 Bydgoszcz, Poland; 2 Department of Analysis and Strategy, Ministry of Health, 00-952 Warsaw, Poland; k.bartolik@mz.gov.pl; 3 Department of Internal Medicine, Pulmonary Diseases and Allergy, Medical University of Warsaw, 02-091 Warsaw, Poland; jacek.nasilowski@wum.edu.pl; 4 VitalAire Home Mechanical Ventilation Centre, 00-180 Warsaw, Poland; 5 Department of Pharmacology and Clinical Pharmacology, Faculty of Medicine, Collegium Medicum, Cardinal Stefan Wyszyński University, 01-938 Warsaw, Poland; 6 2nd Department of Medicine, Department of Pulmonology, Faculty of Medicine, Jagiellonian University Medical College, 30-688 Cracow, Poland; aleksanderkania@interia.pl

**Keywords:** home mechanical ventilation, noninvasive positive pressure ventilation, prevalence, ventilated-assisted patients, chronic respiratory failure, tracheostomy, adult, children

## Abstract

Home mechanical ventilation (HMV) is a dynamically developing field of medicine driven by the increasing number of patients and technological advancements. In Poland, HMV has been financed from public funds since 2004. However, the organization of HMV is still evolving in search of the optimal model of care. The aim of this study was to analyze 11 years of HMV in terms of the number of patients, modes of ventilation, diagnosis and regional prevalence. In retrospective analysis of data reported to the National Health Fund by all health entities providing HMV in Poland in the period from 2009 to 2019, the following variables were included: age, sex, date of commencement, ventilation mode, diagnosis, and place of treatment. The diseases were identified according to the ICD-10 codes. A total of 12,616 patients receiving HMV were reported, including 1221 children (9.7%). The HMV prevalence increased from 2.8 in 2009 to 20/100,000 in 2019. In adults, the highest increase was reported for patients with chronic obstructive pulmonary disease, who accounted for 39% of all HMV users in 2019. The proportion of noninvasive ventilation (NIV) increased from 56% in 2014 to 73% in 2019. We identified significant regional variations in the prevalence of HMV between provinces. The main drivers for HMV development include full reimbursement, the development of hospital NIV centers and the involvement of respiratory physicians in the referral process for HMV.

## 1. Introduction

The origins of home mechanical ventilation (HMV) date back to the 1950s—the polio epidemic [1]. Initially, patients were treated with “iron lungs”, which generated negative pressure around the patient’s chest [2]. Then, the use of positive pressure ventilation via tracheostomy was introduced [3]. The 1980s marked the beginning of noninvasive positive pressure ventilation (NIPPV). The use of long-term ventilation via tracheostomy was gradually limited to a narrow group of patients with bulbar involvement, difficulty evacuating airway secretions and requiring prolonged NIPPV with poor tolerance or efficacy. The possibility of providing HMV by NIPPV opened new therapeutic horizons. It was also found that the progression of ventilatory respiratory failure is a multiannual process, and ventilation support should be introduced early to avoid life-threatening exacerbation and systemic complications of CRF. These observations coincided with scientific advancements in diagnosing sleep-related breathing disorders and related gas exchange impairment. Moreover, new studies were published that demonstrated the efficacy of NIPPV in many patient groups, including chronic obstructive pulmonary disease (COPD) [4] and obesity hypoventilation syndrome (OHS) [5]. Moreover, the ongoing technological advances in HMV equipment have made this treatment method easier to use, more effective and patient-friendly.

The development of HMV varies from country to country. By the end of the 20th century, HMV was already widely used in highly developed countries [6,7]. However, treatment methods, indications and the number of patients varied significantly. The highest prevalence was in France, amounting to 17 per 100,000 inhabitants. These were mainly patients with lung and chest conditions who received NIPPV. In comparison, the prevalence in the Netherlands was 5.6/100,000, but the majority of them suffered from neuromuscular diseases, and approximately 50% required ventilation via tracheostomy [6]. The current prevalence of HMV in Europe is hard to establish based on data in the literature, but it is estimated to range between 20 and 100 users per 100,000 of the population [8].

In Central Europe and in less developed countries in other continents, HMV did not emerge until the beginning of the 21st century. In 2004, Poland was one of the first Central European countries to introduce HMV as a widely available and fully reimbursed form of treatment. Originally, the HMV program was launched to ease pressure on intensive care units (ICUs), which had to take in patients who did not require intensive care but long-term ventilation. This original assumption was a significant milestone in the organization of the home care system for patients requiring ventilation support, giving rise to the emergence of a unique system of care based on regular home visits by qualified medical staff [9]. The growing NIPPV use, the involvement of pulmonologists, and the changing structure of patients contributed to the evolution of HMV care [10]. The aim of this study was to describe the development of HMV in Poland over the second decade of the 21st century.

## 2. Materials and Methods

The aim of the study was to present the development of HMV nationwide and by individual provinces for adult and pediatric populations. It also aimed to demonstrate the dynamics of ventilation methods and groups of diseases underlying the decision to start HMV therapy.

Our study was a retrospective analysis of the National Health Fund (NHF) data reported by all entities providing HMV in Poland between 1 January 2009 and 31 December 2019 as well as comprehensive health care data—on primary health care (PHC), outpatient specialist care (OSC), hospitalization (in hospital and emergency departments), inpatient and outpatient rehabilitation and separately contracted services stored in large population-based administrative medical databases. The NHF is the only public payer for health care services in Poland. Currently, no other Polish institutions provide HMV under private insurance. Thus, it can be assumed that the data from the NHF cover all patients receiving HMV in Poland.

### 2.1. Analyzed Data

The patients were identified in the database of the NHF by PESEL number, i.e., a unique personal identification number assigned to each person at birth. The following data were established for each patient: age, sex, diagnosis, date of commencing and ending HMV care, ventilation mode (from 2014) and provinces of treatment.

This study was a retrospective analysis of the data stored in an official database of the Polish Ministry of Health and National Health Fund. The access to this database is open and not restricted due to privacy policy.

All data were fully anonymous, which was guaranteed by the Polish system of the storage of sensitive personal data. The authors did not have any access to sensitive personal data at any stage of the process of the study.

Data scientists from the Ministry of Health’s Department of Analyses and Strategy analyzed the data and provided a non-identifiable copy of all available information in line with the objectives of the study (to protect patient privacy). Unit data were only processed by analysts who had been authorized to process them. Polish data protection law allows the use of such data, provided it is strictly anonymous, for scientific purposes. Therefore, the patient’s consent and ethical consent were not required and were impossible to obtain.

This study was approved by the Ministry of Health (Project number: POWR.05.02.00-00-0149/15).

### 2.2. Determination of the Cause of Respiratory Failure

The health conditions that served as indications for HMV were identified based on diagnostic codes (International Classification of Diseases, Tenth Revision (ICD-10)) reported by HMV centers. Where a clear diagnosis underlying HMV was impossible to establish due to too general a definition of the disease (e.g., J96—chronic respiratory failure), we took into account comorbidities or information available from other databases and specialist inpatient and outpatient treatment reports throughout patient history. The ICD-10 codes were classified according to the five main groups: neuromuscular diseases (NMDs), restrictive thoracic disorders (RTDs), obstructive lung diseases (OLDs), OHSs and others. The allocation of individual ICD-10 codes to five diagnosis-related groups is shown in Table S1.

The underlying conditions were assigned according to the following rules:

NMD—if at least one NMD code was identified.

RTD—if at least one restrictive lung and/or thoracic disease code was identified without any NMD codes.

OLD—if at least one OLD code was identified and:

- The patient’s ICD-10 code history did not include any NMD, RTD or OHS codes;
- The patient’s ICD-10 code history did not include any NMD or RTD codes, and the OLD code was reported by an outpatient pulmonary clinic or inpatient pulmonary ward.

OHS—if at least one OHS code was identified and did not include any NMD, RTD or OLD codes (or the OLD code was reported by a health facility other than an outpatient pulmonary clinic or inpatient pulmonary ward).

Patients with other diagnoses were automatically assigned to the ‘Other’ group.

Once assigned, the diseases remained unchanged throughout the analysis.

### 2.3. The Prevalence of HMV

The prevalence of HMV is defined as the number of patients receiving HMV per 100,000 of the population in a given year. The number of inhabitants was calculated each year based on demographic data. Adult patients were defined as persons ≥ 18 years of age, and pediatric patients were defined as ≤17 years of age.

### 2.4. Statistical Analysis

The statistical analysis was performed using R software, version 3.6.2 (R Core Team, Vienna, Austria), with IDE RStudio version 1.2.5001 (RStudio Team, Boston, MA, USA). Continuous variables were defined as the means and standard deviation or median, as appropriate.

## 3. Results

### 3.1. Descriptive Characteristics of the Study Subjects

**Figure 1:**
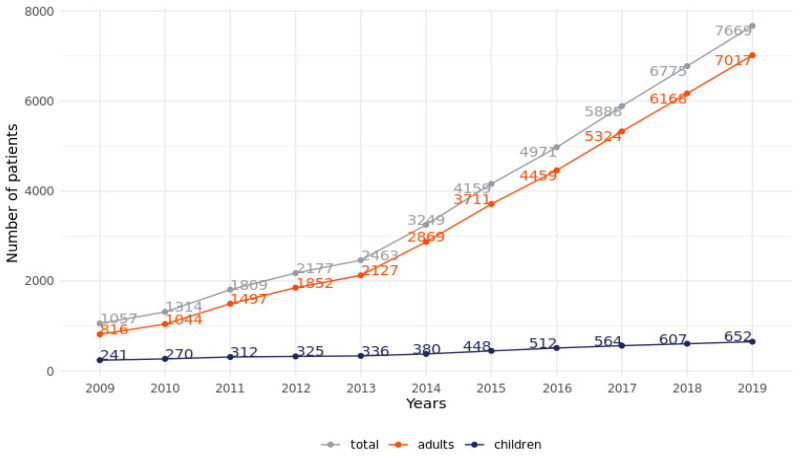
Number of mechanically ventilated patients in 2009–2019.

A total of 12,616 patients receiving HMV were reported, of whom 5541 were women (43.9%), and 1221 were children (9.7%). One thousand and fifty-seven patients were treated in 2009. In the following years, the number of patients continued to increase by approximately 300 per year until 2013, from which point the annual increase reached 800–900 patients. The increase in the number of pediatric patients was less substantial, amounting to approximately 50 patients per year, except for the years 2011–2013, when it was almost flat. Due to the sharp increase in the number of adult patients, there was a steady decrease in the proportion of children from 23% in 2009 to 8.5% in 2019. Detailed data on the total number of patients treated in each year of the study are presented in Figure 1.

### 3.2. Prevalence of HMV

**Figure 2:**
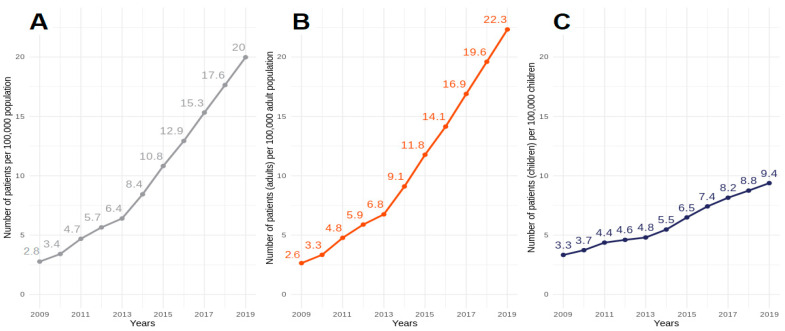
Number of mechanically ventilated patients in Poland per 100,000 of the population in 2009–2019. ((**A**) total, (**B**) adults, (**C**) children).

The prevalence of HMV stood at 2.8 in 2009 and grew consistently by 0.7–1.0 up to 2013, from which point the growth rate began to accelerate, increasing by approximately 2.5 patients. The average rate of increase in the prevalence of HMV in Poland was 2/100,000, with dynamic growth in the adult population, but was much less significant in pediatric patients—1 patient/100,000 children. Ultimately, in 2019, the average prevalence of HMV in Poland was 20/100,000. The prevalence of HMV in Poland across the years broken down into children and adults is presented in Figure 2.

### 3.3. Patient Structure by Age

**Figure 3:**
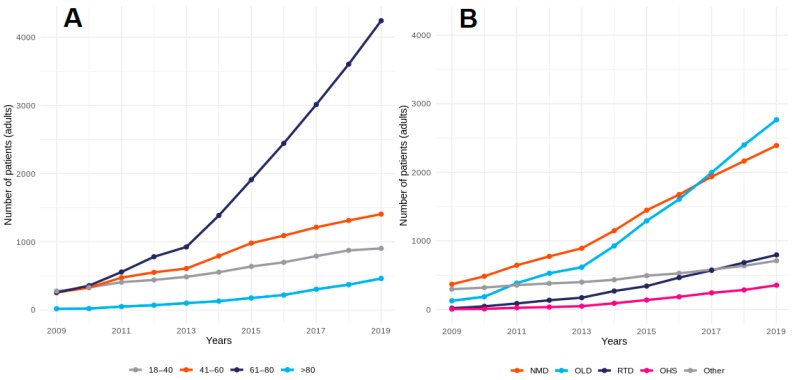
Increase in the number of adult HMV users throughout the years 2009–2019 (**A**) by age group and (**B**) by diagnostic groups.

In 2009, the mean age of adult patients was 49 years (±19.7). Until 2013, the number of patients in all age groups increased at a similar rate. Next, there was a sharp increase in the number of patients aged 61–80 years. In 2019, the mean age was already 62 years (±15.9). There were no changes in the mean age of HMV children, which was 8 years (±5.3). Changes in the age structure of adults are presented in Figure 3A.

### 3.4. Regional HMV Variations

**Figure 4:**
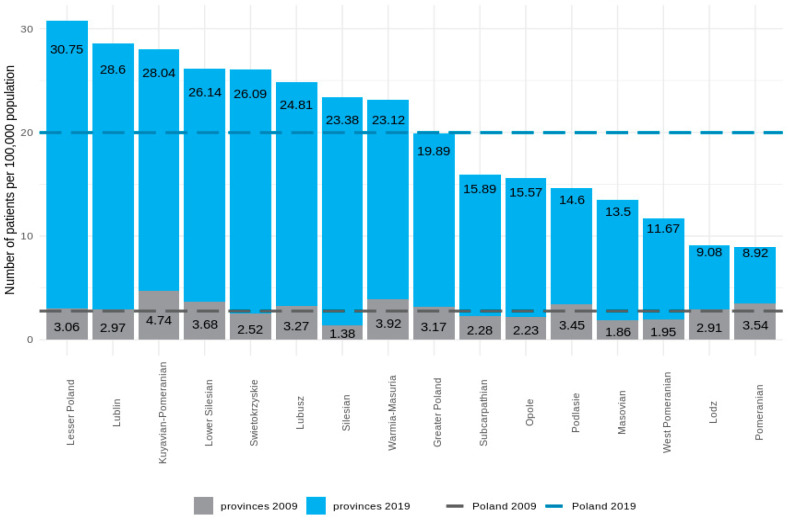
Prevalence of HMV (adults and children combined) in individual provinces in 2009 (grey) and 2019 (blue). The horizontal line represents the average for Poland in 2009 (bottom line) and 2019 (top line).

**Figure 5:**
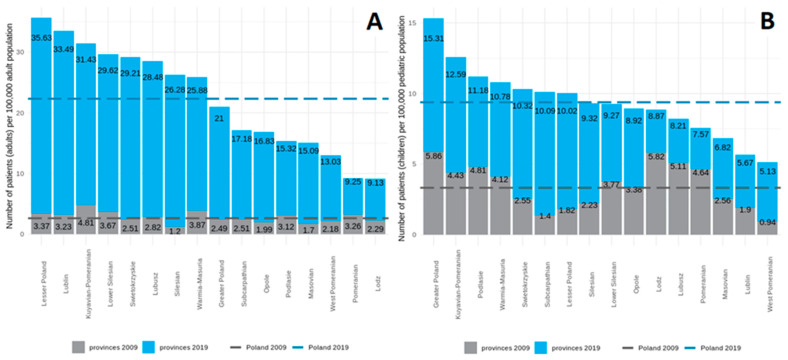
Prevalence of HMV ((**A**) adults, (**B**) children) in individual provinces in 2009 (grey) and in 2019 (blue). The horizontal line represents the average for Poland in 2009 (bottom line) and 2019 (top line).

We found significant differences between provinces in the prevalence of HMV. The highest prevalence was in Lesser Poland—31/100,000 of the population, and the lowest was in Pomerania—9/100,000. In the adult population, differences between provinces were more visible; the highest prevalence was in Lesser Poland—36/100,000 of the population, and the lowest was in Lodz —9/100,000 of the population. In terms of the growth rate, the development of HMV care also varied by region. The highest increase in the prevalence of HMV (1594%) was recorded in Silesia. Detailed data on HMV growth dynamics across provinces in the adult and pediatric populations are presented in Figure 4 and Figure 5.

### 3.5. Diagnostic Groups

Among the five diagnostic groups, the largest group comprised patients with NMD (4792 (38%)), followed by patients with OLD (4358 (35%)), RTD (1251 (10%)), other conditions (1759 (14%)), and OHS (456 (4%)).

In the neurological and neuromuscular group, 182 children (25%) suffered from spinal muscle atrophy, 1792 adults (42%) suffered from amyotrophic lateral sclerosis and 404 (8%) from Duchenne muscular dystrophy. COPD was the most common condition in the OLD group and affected 4176 patients (96%) in that group. The primary cause leading to respiratory failure in 394 patients (31%) in the RTD group was kyphoscoliosis (ICD-10 M40-M41). In the group of patients with other conditions, the most common were congenital and acquired heart diseases, cerebrovascular diseases, and complications following acute, severe respiratory infections (especially in patients in the ICUs). This group also included complications resulting from endocrine conditions, cancers, intoxication due to exposure to chemical, toxic or drug substances, accidents, as well as malformations and upper airway diseases (Pierre Robin syndrome, congenital laryngotracheal stenosis, other congenital laryngotracheal malformations, and vocal cord and larynx paralysis), as well as other congenital syndromes not included in the neuromuscular group.

The distribution of diagnosis-related groups significantly evolved over the reported time period. In 2009, the largest group included patients with NMD, i.e., 45% of all patients. However, due to a dynamic growth in the number of COPD patients, OLD patients were the largest group starting in 2017 (Figure 3B), and in 2019, OLD patients constituted 39% of all patients (Figure S1, Table S2).

### 3.6. Ventilation Methods

**Figure 6:**
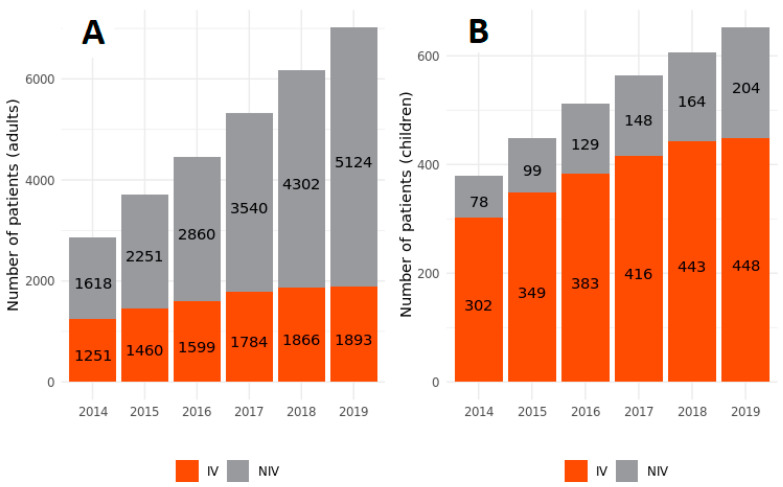
Number of patients mechanically ventilated in the years 2009–2019 acc. to the method of ventilation in adult (**A**) and pediatric (**B**) populations.

The most common mode of ventilation in adults was NIPPV, with prevalence rates gradually increasing from 56% in 2014 to 73% in 2019 (Figure 6, Table S3). Important regional variations were observed in NIPPV prevalence—from 42% in Pomerania to 81% in Kuyavian-Pomerania. Detailed data representing the rate of NIPPV usage across the country in adult and pediatric populations are shown in Table S3.

**Figure 7:**
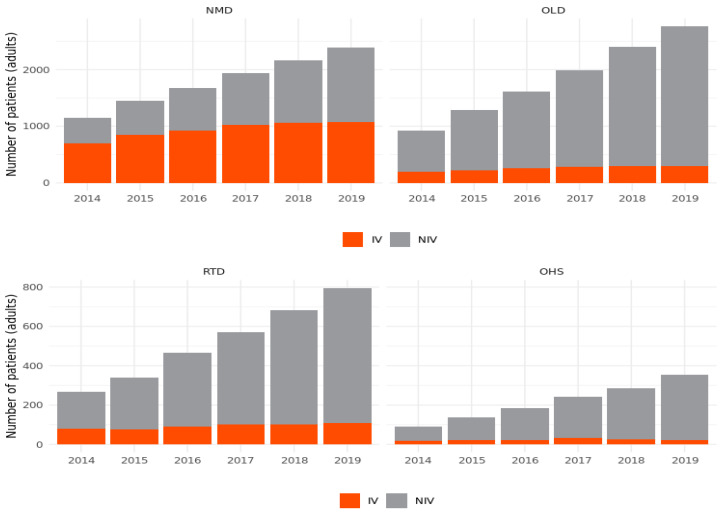
Number of mechanically ventilated patients (adults) acc. to condition and the method of ventilation in the years 2014–2019.

Increased prevalence in NIPPV was observed in all diagnostic groups. However, the rising trend was the slowest in the NMD: it rose from 39% in 2014 to 65% in 2019. Given the increasing number of patients receiving NIPPV, the percentage of invasive ventilation support gradually decreased in the respective diagnostic groups (Figure 7, Table S4). Although the number of children receiving NIPPV has been increasing over the last 6 years of observation, invasive ventilation is still the most common method of ventilation in the pediatric population (66%) (Figure 6).

## 4. Discussion

To the best of our knowledge, this paper is the first to describe the usage of HMV within a single, large European country in such a comprehensive and reliable manner. It owes these qualities to the methodology we used to collect the data. The method consisted of obtaining complete data relating to HMV in Poland from the NHF, which is the only institution to finance treatment in Poland. The research conducted so far to assess the use of HMV was typically based on survey data. This methodology was also applied in a broadly discussed study describing the patterns of use of HMV in several European countries, known as EUROVENT [6]. The EUROVENT data then became the point of reference for successive analyses outlining the usage of HMV in different countries or regions [11]. The study comprised 13 Western European countries plus Poland as the only representative of Central and Eastern European countries. The data clearly indicated that there was a significant difference between Poland, where HMV was used by 0.1 patients per 100,000 of the population, and the other countries, where the prevalence rate was 6.6. In addition to that, NIPPV was also rare in Poland, and the majority of mechanically ventilated patients suffered from NMD. The data reported in the EUROVENT study were from the years 2001/2002, i.e., when HMV was not yet reimbursed in Poland. It was not until 2004 that the National Health Fund started funding HMV in Poland, which also marked a systematic growth in the use of HMV.

Data from 2008, also based on complete reports from the NHF, show that the average prevalence of HMV was 2.2: 60% of patients received an invasive ventilation support [9]. Nasilowski et al. published a detailed analysis of the patterns of use of HMV in Poland in the years 2000–2010 [10]. The data used in their study were derived from a survey conducted by the largest HMV centers in Poland. According to the survey, HMV was systematically developing not only in terms of the number of patients, but also in terms of a gradual decrease in the percentage of patients receiving invasive ventilation and suffering from NMD—a shift towards patients with lung and chest diseases. Our work is a continuation of the study by Nasilowski et al., as it represents a further development of HMV in Poland in the years 2009–2019.

The prevalence of HMV in Poland increased from 2.8 to 20/100,000 in 2009–2019. However, the rate of increase was not homogeneous. If we look at the chronology, two points in time are critical: 2014 and 2017. From 2014, the number of HMV patients was significantly increasing due to several factors. First, the Minister of Health enforced a new regulation of NIPPV in hospitals. Second, the NHF started funding NIPPV provided in a hospital setting. Third, according to the regulation, pulmonologists were allowed to qualify patients for HMV (previously, only anesthesiologists were authorized to do so). Fourth, the funding of NIPPV and invasive ventilation at home was diversified, which allowed more patients to be treated within the same funding program. Fifth, the provision of systematic training to healthcare professionals across the country on NIPPV and HMV was organized by the Polish Respiratory Society. Sixth, breakthrough study results were published, providing evidence behind the effectiveness of HMV in COPD patients [4]. In 2017, for the first time, the number of patients suffering from OLD receiving HMV was higher than the number of patients with NMD. This shift was caused by the launch of a healthcare program called the “2016–2019 National Chronic Lung Disease Mortality Rate Reduction Programme: Noninvasive Mechanical Ventilation at Hospitals”—POLVENT—by the Ministry of Health [12]. A total of 36 respiratory high-dependency care units were opened across Poland dedicated to NIPPV in the treatment of acute respiratory failure [12]. At the same time, these units qualified eligible patients for HMV. In the same year, Murphy et al. argued that a relatively prompt (2–4 weeks after exacerbation) qualification for HMV of patients following COPD exacerbation resulted in longer periods until the next exacerbation requiring hospitalization [13].

Reliably comparing HMV prevalence in different countries is not an easy task since the reported data come from different periods and have been derived using different methodologies. However, a reference to a Canadian study [14], the closest in terms of methodology, shows that in a comparable period, i.e., around 2012, HMV prevalence in Canada and Poland was similar, i.e., 5.0 and 5.65/100,000 of the population, respectively. However, in Canada, the overall prevalence of HMV was estimated based on the results from only one region (Ontario) [14]. An improved ratio of noninvasive to invasive mechanical ventilation is also the effect of HMV development and the involvement of pulmonologists. This shift, which started in 2014, is evidently in favor of NIPPV.

The development of HMV and inclusion of new patients (mainly with COPD) in NIPPV therapy also helped change the population distribution of patients receiving HMV by age. Over the first decades following its introduction, HMV was used primarily in younger patients (49 years of age on average) and due to NMD. By qualifying more COPD patients, the average age increased to 62.

Our observations show that there are significant inequalities regarding access to HMV in Poland, despite a generally uniform healthcare system. Interestingly, regions that were pioneers in the past are still leaders today. This disproportion is due mainly to the commitment of local HMV leaders.

Among all the studied groups of patients receiving HMV, neither increased quite as much in number as the COPD group. In this group, the prevalence of NIPPV was also the highest, i.e., 89% in 2019. Potential explanations are as follows: 1. new evidence-based data emerging from clinical studies [4,13], which shift in paradigm regarding HMV indications for COPD patients, 2. raising awareness among doctors of the possibilities of treating COPD, and 3. improving prognosis.

Although the number of children receiving HMV has significantly increased globally over the last decades [15,16,17,18,19], the prevalence in the pediatric population has not been studied sufficiently. Studies analyzing the prevalence of HMV tend to include children in the adult population. Only a few papers considered children separately, including a South Korean study [20], which is based on administrative data obtained from the National Health Insurance Service in 2016 and which estimates the HMV prevalence rate of 4.4/100,000 children [20]. Goodwin et al. discussed HMV prevalence in the southwest region of Great Britain in the last 15 years on the basis of a retrospective overview of all patients under 18 years of age from a local database and hospital administration systems in the period from 1994 to 2009 [21]. The study shows that the prevalence rate increased during that period from 0.2 to 6.7 per 100,000 children in 2009. In our study, the HMV prevalence rate in children was 3.3 per 100,000 children in 2009 and 9.4 per 100,000 children 11 years later, indicating a significant increase in HMV in the pediatric population. Similar to trends in the adult population, regional disparities were also significant over the last 11 years (in 2019, the difference ranged from 5.1 to 15.3 per 100,000 children). Moreover, NIPPV is becoming increasingly prevalent in children. According to a study by Shuk-Kuen Chau et al., invasive mechanical ventilation was used in 26% of the studied children [22]. This percentage was comparable in Great Britain (22%) [15] and Switzerland (31%) [23]. An even lower percentage of invasive mechanical ventilation in children was noted in Canada (17%) [24]. Invasive ventilation was predominant in the Polish population, amounting to two-thirds of the pediatric population in 2019. These differences may result from a small number of pediatric centers in Poland qualifying children to NIPPV at home, inclusion criteria, or the prevalence rate of a given disease.

Our study, similar to other administrative data-based studies, has some limitations. First, it does not include sufficient clinical data, which makes the assessment of patient HMV qualification and adherence difficult. Moreover, we could not analyze such vital outcomes as improvement in the quality of life [25], decrease in the exacerbation rate, or improvement in survival. The lifespan of HMV users, depending on diagnosis, should be a subject of further research. Second, in many cases, the characteristics of primary diagnoses leading to chronic ventilation have not been verified by practitioners due to incomplete reports, and therefore might be the source of inaccuracy. This applies in particular to other conditions—we could not identify diagnoses on the basis of administrative data regarding specialist outpatient or hospital healthcare in patients’ records.

We believe that our analysis may help inform economic optimization strategies regarding the further development of HMV. It may also be used to help guide other countries in the development of HMV and show them how systemic solutions affect the growth of this treatment. A national register of HMV users and the existing technology enabling monitoring of patient adherence and effectiveness of therapeutic recommendations could help.

## Data Availability

The data that support the findings of this study are available from the Ministry of Health’s Department of Analyses and Strategies in Poland. The data are protected under the Protection of Privacy Act. Access to the data will require formal permission by the Data Stewards within the Ministry of Health.

## Supplementary Materials

The following supporting information can be downloaded at: https://www.mdpi.com/article/10.3390/jcm11082098/s1, Figure S1: Percentage of adult patients mechanically ventilated acc. to condition in the years 2009–2019; Table S1: The allocation of individual ICD-10 codes to five diagnosis-related groups; Table S2: Number (percentage) of adult patients mechanically ventilated acc. to condition in the years 2009–2019; Table S3: Percentage of mechanically ventilated patients (adults and children) acc. to voivodeships and the method of ventilation in 2014 and 2019; Table S4: Number (percentage) of mechanically ventilated adult patients acc. to condition and the method of ventilation in the years 2014–2019.
