# Supplementary material for: Development of Home Mechanical Ventilation in Poland in 2009–2019 Based on the Data of the National Health Fund
Source: J Clin Med. 2022 Apr 9;11(8):2098. doi: 10.3390/jcm11082098 (PMC9032651; doi:10.3390/jcm11082098)
Supplement: Supplementary file 1 [file jcm-11-02098-s001.zip › jcm-1606051-supplementary.pdf]

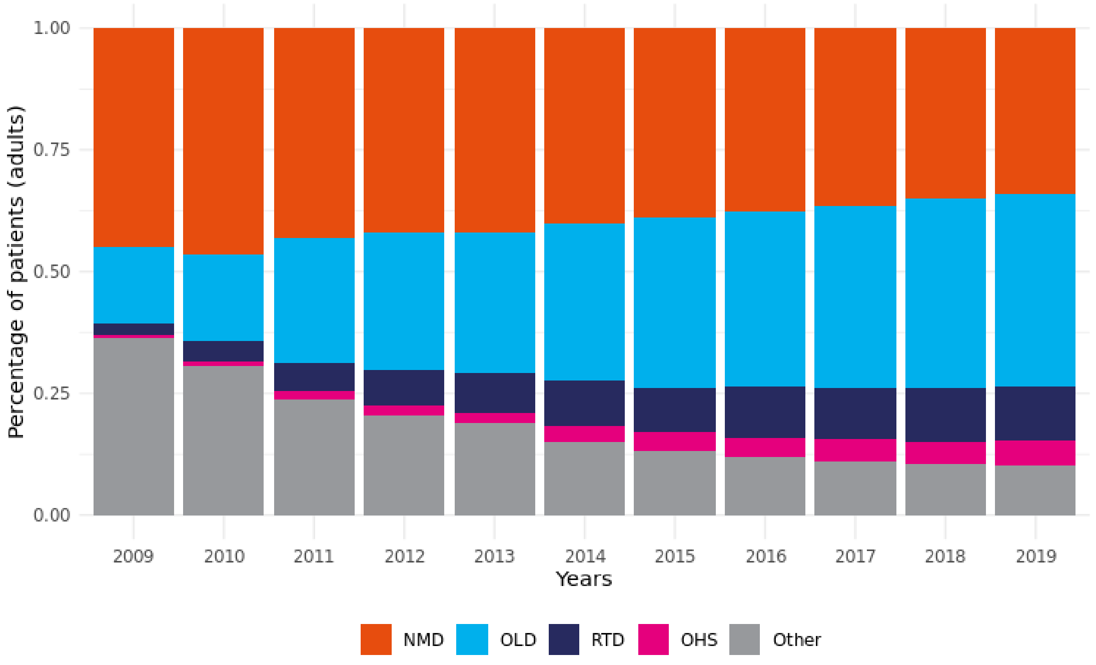

Figure S1. Percentage of adult patients mechanically ventilated acc. to condition in the years 2009-2019.

Table S1. The allocation of individual ICD-10 codes to five diagnosis-related groups.

Diagnostic group	Diseas e grup	Subtypes/disease	ICD-10
**I. Neuromuscular diseases (NMD) and central nervous system disorders**	Spinal muscular atrophy (SMA) and related syndromes		G12
		SMA I, SMA II, SMA III, SMA IV	G12.0; G12.1
		**Amyotrophic lateral sclerosis (ALS)**	G12.2
		Other spinal muscular atrophies and related syndromes	G12.8
		Spinal muscular atrophy, unspecified	G12.9
	Post-polio syndrome		G14
	Phrenic nerve paralysis due to birth injury		P14.2
	Myasthenia gravis and other myoneural disorders		G70
		Myasthenia gravis	G70.0
		Congenital and developmental myasthenia	G70.2
	Primary disorders of muscles		G71
		**Muscular dystrophy (MDs)**<br>Duchenne muscular dystrophy (DMD)<br>Becker Muscular dystrophy (BMD)<br>Limb-girdle MD	G71.0
		**Myotonic Dystrophies**<br>Type 1 (Curschmann-Steinert)<br>Dystrophia myotonica [Steinert]	G71.1
		**Congenital myopathies**<br>Myotubular, nemaline<br>Central core disease	G71.2
		**Mitochondrial** myopathy, not elsewhere classified	G71.3
		Other primary disorders of muscles	G71.8
		Primary disorder of muscle, unspecified	G71.9
		**Myopathy in metabolic diseases:**	

		Glycogenosis type II (Pompe disease, adult form)<br>Glycogen storage disease lipid storage disorders	E74.0<br>E75.2
	Acquired neuromuscular diseases		
		Critical illness associates myopathy<br>Guillain-Barré syndrome	G72.8<br>G61.0
	Congenital malformations		
		Congenital malformations of the musculoskeletal system, not elsewhere classified	Q79
		Congenital malformation syndromes predominantly associated with short stature (Prader-Willi Syndrome)	Q87.1
		Chromosomal abnormalities, not elsewhere classified<br>Down syndrome<br>Edwards syndrome and Patau syndrome	 Q90<br>Q91
	Other neuromuscular diseases		
		Parkinson disease	G20
		Multiple sclerosis	G35
		Cerebral palsy	G80
	Central nervous system disorders		
		Arnold-Chiari malformation	Q07.0
		Central nervous system trauma	S06
		Myelomingocele	Q01, Q05
		Spinal cord traumatic injuries	S14
**II. Restrictive lung and thoracic diseases (RTD)**	Kyphoscoliosis and chest deformities		
		Kyphoscoliosis	M41
		Kyphosis	M40
		Other deforming dorsopathies	M43
		Bechterew syndrome	M45
		Acquired deformity of chest and rib	M95.4
		Postprocedural respiratory disorders, not elsewhere classified	J95
	Restrictive lung diseases		
		Sequelae of respiratory and unspecified tuberculosis	B90.9
		Other interstitial pulmonary diseases	J84
**III. Obstructive lung diseases (OLD)**			
	COPD		J44
	Cystic Fibrosis		M84
	Bronchiectasis		J47
**IV. Obesity hypoventilation syndrome (OHS)**			E66.2
**V. Other**			
	Upper airway disorders		
	Complications of infectious pneumonias		
	Congenital and acquired pulmonary diseases such as pulmonary hypoplasia and bronchopulmonary dysplasia		
	Cardiovascular disorders		
	Cerebrovascular disorders		
	Others		

Table S2. Number (percentage) of adult patients mechanically ventilated acc. to condition in the years 2009-2019.

Year	Neuromuscular diseases	Obstructive lung diseases	Restrictive lung and thoracic diseases	Obesity hypoventilation syndrome	Other
2009	368 (45%)	127 (16%)	19 (2%)	6 (1%)	296 (36%)
2010	484 (46%)	186 (18%)	46 (4%)	9 (1%)	319 (31%)
2011	645 (43%)	384 (26%)	88 (6%)	25 (2%)	355 (24%)
2012	774 (42%)	528 (28%)	135 (7%)	35 (2%)	380 (20%)
2013	892 (42%)	616 (29%)	172 (8%)	48 (2%)	399 (19%)
2014	1149 (40%)	927 (32%)	269 (9%)	91 (3%)	433 (15%)
2015	1447 (39%)	1292 (35%)	341 (9%)	138 (4%)	493 (13%)
2016	1675 (38%)	1607 (36%)	465 (10%)	185 (4%)	527 (12%)
2017	1936 (36%)	1995 (38%)	570 (11%)	242 (4%)	581 (11%)
2018	2164 (35%)	2398 (39%)	684 (11%)	285 (5%)	637 (10%)
2019	2391 (34%)	2766 (39%)	796 (11%)	354 (5%)	710 (10%

Table S3. Percentage of mechanically ventilated patients (adults and children) acc. to provinces and the method of ventilation in 2014 and 2019.

Provinces	adults				children			
	2014		2019		2014		2019	
	IV	NIV	IV	NIV	IV	NIV	IV	NIV
Lower Silesian	26%	74%	21%	79%	71%	29%	74%	26%
Kuyavian-Pomeranian	32%	68%	19%	81%	59%	41%	57%	43%
Lublin	37%	63%	23%	77%	55%	45%	67%	33%
Lubusz	51%	49%	20%	80%	88%	12%	53%	47%
Lodz	45%	55%	35%	65%	69%	31%	62%	38%
Lesser Poland	38%	62%	21%	79%	94%	6%	82%	18%
Masovian	44%	56%	27%	73%	83%	17%	52%	48%
Opole	45%	55%	25%	75%	83%	17%	79%	21%
Subcarpathia	62%	38%	43%	57%	87%	13%	59%	41%
Podlasie	89%	11%	52%	48%	87%	13%	83%	17%
Pomeranian	82%	18%	58%	42%	85%	15%	83%	17%
Silesian	37%	63%	21%	79%	70%	30%	64%	36%
Swietokrzyskie	35%	65%	23%	77%	78%	22%	62%	38%
Warmia-Masuria	43%	57%	26%	74%	84%	16%	64%	36%
Greater Poland	65%	35%	38%	62%	87%	13%	84%	16%
West Pomeranian	48%	52%	48%	52%	82%	18%	40%	60%
**Poland** (total)	**44%**	**56%**	**27%**	**73%**	**79%**	**21%**	**69%**	**31%**

Table S4. Number (percentage) of mechanically ventilated adult patients acc. to condition and the method of ventilation in the years 2014-2019.

**Condition**	**Method of ventilation**	**2014**	**2015**	**2016**	**2017**	**2018**	**2019**
Neuromuscular diseases	NIV	450 (39%)	603 (42%)	757 (45%)	918 (47%)	1100 (51%)	1315 (55%)
Neuromuscular diseases	IV	699 (61%)	844 (58%)	918 (55%)	1018 (53%)	1064 (49%)	1076 (45%)
Obstructive lung diseases	NIV	728 (79%)	1068 (83%)	1353 (84%)	1712 (86%)	2100 (88%)	2472 (89%)
Obstructive lung diseases	IV	199 (21%)	224 (17%)	254 (16%)	283 (14%)	298 (12%)	294 (11%)
Restrictive lung and thoracic diseases	NIV	190 (71%)	266 (78%)	375 (81%)	467 (82%)	584 (85%)	688 (86%)
Restrictive lung and thoracic diseases	IV	79 (29%)	75 (22%)	90 (19%)	103 (18%)	100 (15%)	108 (14%)
Obesity hypoventilation syndrome	NIV	72 (79%)	117 (85%)	161 (87%)	209 (86%)	260 (91%)	333 (94%)
Obesity hypoventilation syndrome	IV	19 (21%)	21 (15%)	24 (13%)	33 (14%)	25 (9%)	21 (6%)
